# Exploring cross-protective effects between cold and immune stress in *Drosophila melanogaster*[Fn FN1]

**DOI:** 10.1051/parasite/2023055

**Published:** 2023-12-12

**Authors:** Jakob Wiil, Jesper Givskov Sørensen, Hervé Colinet

**Affiliations:** 1 Université de Rennes, CNRS, ECOBIO [(Écosystèmes, biodiversité, évolution)] – UMR 6553 263 AVE du Général Leclerc 35000 Rennes France; 2 Department of Biology, Aarhus University Ny Munkegade 8000 Aarhus C Denmark

**Keywords:** Cross talk/tolerance, Stress response, Biotic stress, *Drosophila melanogaster*, *Turandot* genes

## Abstract

It is well established that environmental and biotic stressors like temperature and pathogens/parasites are essential for the life of small ectotherms. There are complex interactions between cold stress and pathogen infection in insects. Possible cross-protective mechanisms occur between both stressors, suggesting broad connectivity in insect stress responses. In this study, the functional significance of these interactions was tested, as well as the potential role of newly uncovered candidate genes, *turandot*. This was done using an array of factorial experiments exposing *Drosophila melanogaster* flies to a combination of different cold stress regimes (acute or chronic) and infections with the parasitic fungus *Beauveria bassiana*. Following these crossed treatments, phenotypic and molecular responses were assessed by measuring 1) induced cold tolerance, 2) immune resistance to parasitic fungus, and 3) activation of *turandot* genes. We found various responses in the phenotypic outcomes according to the various treatment combinations with higher susceptibility to infection following cold stress, but also significantly higher acute cold survival in flies that were infected. Regarding molecular responses, we found overexpression of *turandot* genes in response to most treatments, suggesting reactivity to both cold and infection. Moreover, maximum peak expressions were distinctly observed in the combined treatments (infection plus cold), indicating a marked synergistic effect of the stressors on *turandot* gene expression patterns. These results reflect the great complexity of cross-tolerance reactions between infection and abiotic stress, but could also shed light on the mechanisms underlying the activation of these responses.

## Abstract (fr)

Il est établi que les stress environnementaux et biotiques, tels que la température et les agents pathogènes ou parasites, sont essentiels à la survie des petits ectothermes. Il existe des interactions complexes entre le stress froid et l’infection par des pathogènes chez les insectes. Des mécanismes de protection croisée sont possibles entre les deux facteurs de stress, suggérant une grande connectivité entre ces réponses. Dans cette étude, ces interactions ont été testées, ainsi que l’implication potentielle de nouveaux gènes candidats, les gènes *turandot*. Cela a été fait via un ensemble d’expériences factorielles exposant des mouches *Drosophila melanogaster* à différents types de stress froid (aigu ou chronique) couplés à des infections par le champignon entomoparasite *Beauveria bassiana*. Suite à ces traitements croisés, les réponses phénotypiques et moléculaires ont été analysées en mesurant 1) la tolérance induite au froid, 2) la résistance immunitaire à l’infection pathogène et 3) l’activation des gènes *turandot*. Les résultats phénotypiques ont montré des réponses variées selon les différentes combinaisons de traitements, avec une plus grande susceptibilité à l’infection suite à un stress froid, mais aussi une survie au stress froid aigu significativement plus élevée chez les mouches infectées. Pour les réponses moléculaires, nous avons trouvé une surexpression des gènes *turandot* en réponse à la plupart des traitements, suggérant une réactivité à la fois au froid et à l’infection. De plus, les expressions maximales ont été distinctivement observées dans les traitements combinés (infection plus froid), indiquant un effet synergique marqué des facteurs de stress sur l’expression des gènes *turandot*. Ces résultats reflètent la complexité des réactions de tolérance croisée entre infection et stress abiotique, et pourraient également éclairer les mécanismes sous-jacents de l’activation de ces réponses.

## Introduction

Cold tolerance is considered a major factor determining the distribution of insect species [1, 30]. The degree and severity of adverse effects following cold stress are highly variable with both duration and intensity. For the small ectotherms that are considered chill-susceptible, even temperatures above the freezing point of extracellular fluid cause cellular disruption and damage [4, 13]. An example of this can be found in the variable impact of cold stress in *Drosophila melanogaster*, where brief intense exposure to cold causes cold shock injury such as the initiation of apoptosis [73] or membrane phase transition [15], while longer cold exposures appear to cause disruptions in the ion and water balance, effectively inducing desiccation-like symptoms [43, 46, 62]. Ectothermic organisms have evolved intra-generational plastic responses to alleviate and adapt to stressful conditions [27, 67]. For instance, thermal hormetic effects, typically referred to as hardening, are observed for both heat and cold [10]. For example, mild thermal stress in young *D. melanogaster* induced increased thermal tolerance to severe thermal stress throughout the entire life-span of the flies [35].

Although the direct effect of thermal stress on an organism is profound, it is not the only way temperature can interact and affect biological systems. A wide array of secondary interactions between the responses to temperature and other stressors have been uncovered. One example is the interaction between cold stress and biotic (infection) stress [20]. Interactions can take various forms, the simplest of which concerns the additive negative effect of each stressor. Yet, hormetic beneficial plastic responses where exposure to a moderate stress of one kind protects against another kind of stress also exist [57, 65]. This concept is known as cross-talk, where activation of a shared signaling pathway results in induction of downstream stress protection pathways, or cross-tolerance, in which the signaling pathways against different stressors are not shared but the physiological changes resulting from activation of response to one stress leads to protection against another [57, 62, 65].

Recent research focusing on insect tissue and cellular response on a molecular level has shown common molecular events and cellular pathways between immune stress response and other stress responses hinting at cross-talk/tolerance mechanisms [12, 69]. The most notable of these is arguably the effect of cold stress inducing tolerance to pathogenic infection in insects [59, 62]. Studies testing this concept of cross-protective responses in *D. melanogaster* have found mild cold stress activation of immune genes to produce pathogenic tolerance in the organism [62]. For example, Le Bourg [35] found that mild cold stress had a positive effect on resistance to infection by the entomoparasitic fungus *Beauveria bassiana*, significantly increasing longevity of infected flies compared with other stressors, like heat and hyper-gravity. Conversely, studies have also investigated the inverse response of immune-induced cold tolerance in *D. melanogaster.* However, some evidence seems to suggest a negative interaction – for example, bacterial infection increasing the time taken to recover from chill coma in *D. melanogaster* (thereby reducing cold tolerance) [40]. The activation of immunity depends on the type of cold stress or cold injury. For instance, transcriptomic response to supercooling was dominated by upregulation of immune response pathways, while the response to freezing contained only few elements of immune response [66]. It seems that interactions between cold stress and immunity, whether positive or negative, are context-dependent and further studies are therefore needed to clarify these under-investigated phenomena.

Cold × immune interactions are known across a diverse range of orders including Lepidoptera, Hymenoptera and Heteroptera [31, 49, 72]. Multiple, non-mutually exclusive hypotheses of their evolutionary and adaptive heritage have been proposed. For non-adaptive or deleterious theories, one concerns the implication of cold exposure activating common pathways shared with the immune response thereby leading to non-adaptive immunity. However, as recovery from cold stress is already energetically costly, this secondary activation should be selectively advantageous [45, 62]. Another theory pertains to the negative synergistic effect cold stress seems to have on immune capabilities, with some studies finding cold stress to heighten the susceptibility to infection [7, 20, 33, 52]. As for adaptive responses, immune responses could be required to repair damage following cold exposure [59, 62]. An example could be damage to gut tissue, allowing gut flora or bacterial cell components (*e.g.*, pieces of cell membranes/walls) to enter the hemocoel, thereby triggering an antimicrobial response. Consequently, there may have been selection for an anticipatory activation of immunity due to the association between cold and such wounding [44, 62]. Recently it was found that leak of fluorescent bacteria did not occur in the gut of cold stressed locusts [18]; however, other leaking immunogenic components such as peptidoglycan and lipopolysaccharide may also trigger antimicrobial response in the host. Another hypothesis revolves around the adaptive nature of pre-emptive immune activation evolving in connection with a mismatch in the thermal performance between pathogen and host organisms, providing protection against cold-active pathogens [20, 62]. In this study, it is conjectured that the onset of cold (and therefore cold stress) can be used as “pre-adaptation” to activate the immune system in preparation for the seasonal drop in temperature [7, 57]. Indeed, while winter is often a period of repression (*e.g.*, of metabolism or activity) for hosts, pathogens on the other hand can remain cold active and threaten insect survival over winter. For instance, most strains of *Beauveria bassiana*, an entomoparasitic fungus, remain active at low temperature [21, 64]. Hence, cold-activation of immunity may be useful since insects may be exposed to infection risk throughout the overwintering period. This has been echoed more widely in recent years, in line with current expansion of knowledge relating to the importance of cold stress as a source of environmental information in insect eco-immunology [20, 59, 62].

As interest in the cross-protective effects of cold and immunity has grown, a family of recently discovered immune-related genes has been proposed as a candidate for this phenomenon. The *turandot (tot)* gene family contains 8 different genes formerly identified as infection-responsive genes in *D. melanogaster* [17]*.* The precise function of *turandot* genes is still unknown but *tot* genes have been linked to inflammation processes resulting from microbial invasion or tissue damage [63]. Some studies have uncovered similarities between *tot* genes and *HSP* genes, primarily in the overlapping patterns of activation, indicating that *tot* genes could be part of a general stress response mechanism [3, 16, 51]. This is even further signified by *tot* genes being activated/upregulated in response to a multitude of stressors including heat stress, irradiation, infection, dehydration, oxidative agents, and mechanical stress [3, 6, 16, 17, 48, 59]. In particular, *tot* genes have been found to be activated by several kinds of cold stress treatments, including chronic cold, acute cold, and repeated exposure treatments, making the cold activation of *tot* genes specifically well established [26, 48, 59, 75]. In a recent study, a *tot* gene was among the most upregulated genes in cold acclimated *Drosophila suzukii* flies [19]. As a result, these cold-activated immunity-related genes are potential candidates to explain the interaction between cold and immunity.

The study of cross-protective mechanisms in insects is still in its early stages. Knowledge gained is of ever-increasing importance for our understanding of potential pre-adaptive plastic capabilities of small ectotherms in a rapidly changing world [57]. For example, with seasonality and temperature expected to become increasingly variable with changing climates, overwintering insects can experience mismatches or disturbances in the way cold is used to gather information about needed immune responses [20]. This constitutes a mere fraction within the wider context of a larger pivotal change in the effect that global warming temperatures are expected to have on immune functions in insects as a whole, making the advancement of knowledge in this field of the upmost important for future scientific research [47, 68].

The aim of this study was to investigate the cross-protective aspects of insect immune and low temperature responses with specific focus on the functional significance of the candidate gene family *Turandot*. We aimed to expand our knowledge both of this under-investigated gene family and of the general stress response and cross-protective mechanisms in insects. To experimentally test these concepts, three individual sets of experiments were set up to investigate the three branches of the relationship between thermal and entomoparasitic stress and the potential cross-protective effect produced in the subsequent induced stress responses. Two set of experiments were created to test both actual induced immunity following exposure to cold stress [cold × immune], and actual cold tolerance following exposure to entomoparasitic stress [immune × cold]. Additionally, temporal expression profiles were analyzed for four *tot* genes, following different combinations of cold and infection stress.

We tested the following hypotheses: 1) In response to cold treatments (chronic and/or acute stress), *D. melanogaster* flies will induce an immune response resulting in a higher survival rate following infection with the parasitic fungus *B. bassiana*. This would imply the existence of an actual cross-protective effect between the two stressors, triggered by cold; 2) In response to an infection by *B. bassiana*, *D. melanogaster* flies will induce an adaptive cold protective response, resulting in a quicker capacity to recover from cold stress and higher survival in infected flies compared to control flies; 3) The temporal dynamics of the immune and cold tolerance following treatment will be concurrent with the upregulation of *tot* genes.

## Materials and methods

### Husbandry and rearing

We used an outbred laboratory population of *D. melanogaster* established from wild individuals collected in September 2015 in Rennes, Brittany (France). Fly stocks were maintained, and all experiments conducted at 25 °C and 70% relative humidity (12 h light:12 h darkness) on standard food comprising inactive brewer’s yeast (MP Bio 029 033 1205, MP Bio, 80 g/L), sucrose (50 g/L), agar (Sigma-Aldrich A1296, 10 g/L), and supplemented with anti-mold Nipagin (Sigma-Aldrich H5501; 10% 8 mL/L) and propionic acid (10 mL/L; 0.01 M). Only adult female flies were used in the experiments to avoid any inter-sex variance affecting the outcomes. Adult female flies awaiting treatment were kept in bottles containing food medium at a density of ~ 100 flies/bottle. Likewise, all development of new generations was done using the same food medium recipe. Adult flies were changed to fresh food at approximately 2-day intervals to avoid detrimental environmental effects on food quality at 25 °C and every 3 days at 15 °C.

To generate flies for the experiments, groups of 15 mated females were allowed to lay eggs in 100 mL rearing bottles during a restricted period of (less than 6 h) under laboratory conditions. This controlled procedure allowed larvae in bottles to develop under uncrowded conditions. The collection and sexing of all flies were done by eye, without CO_2_ anesthesia, using morphological differences expected between sexes. Flies awaiting treatment were placed in bottles containing food medium at approx. 100 females per bottle. Flies from several bottles were mixed in this process to randomize and therefore avoid any potential bottle effects. Experimental females were left to age on food for 5 days (food was changed every two days) under standard conditions before they were assigned to the treatments.

### Cold × infection experiments

**Figure 1:**
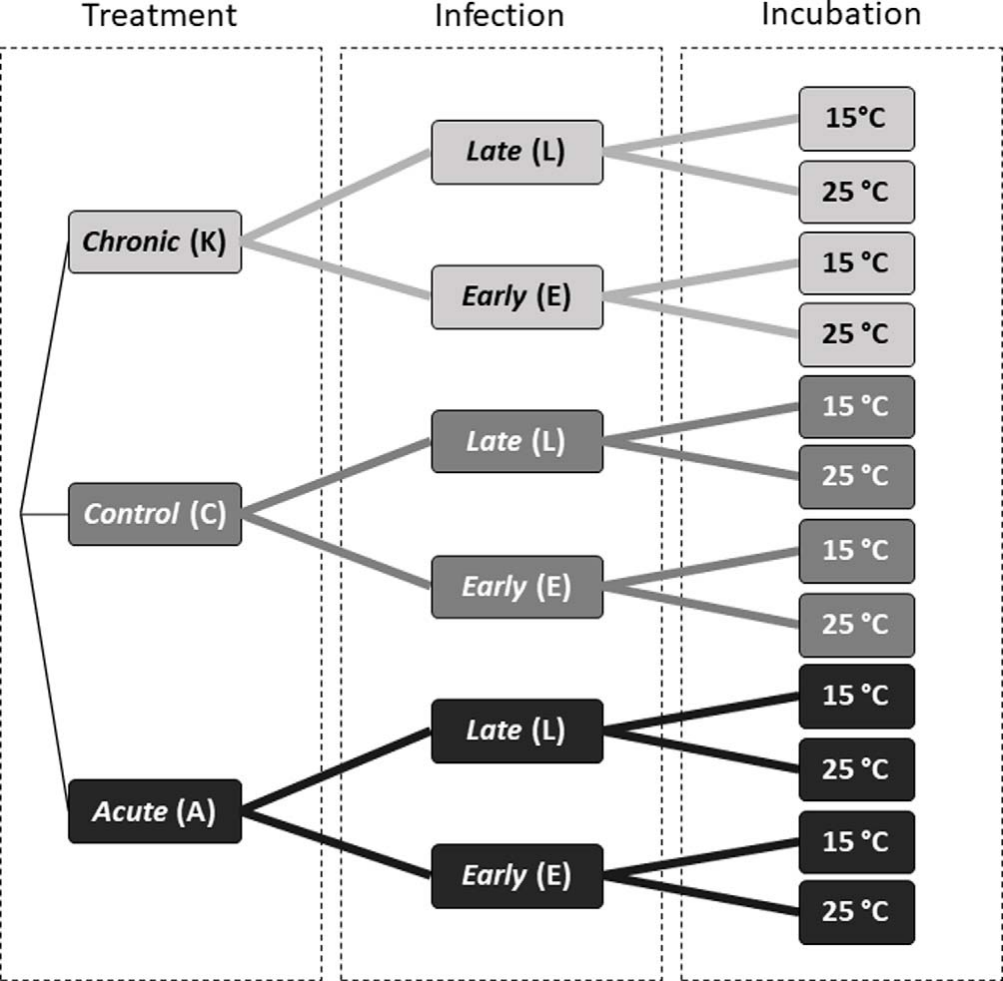
Graphical representation of the twelve combinations of treatments and color codes resulting from three fully crossed factors: 1) cold treatment, 2) infection time, and 3) incubation temperature. See Materials and methods for full descriptions of treatments.

In these experiments, we sought to elucidate the potential cross-protective effect of two different cold treatments on the pathogenic resistance in *D. melanogaster* following infection with *B. bassiana.* We produced a full factorial design with three interacting factors: cold pre-treatment (3 levels), infection time following the cold treatment (2 levels) and the incubation temperature of infected flies (2 levels), resulting in 12 conditions. The full factorial design is illustrated in Figure 1. For cold treatment, the three levels were *chronic* cold stress (K), *acute* cold stress (A), or an unexposed *control* (C) (see below). Two additional layers of sub-groups within these treatments were created. First concerning the infection time following the cold treatment, the two levels were *early* (E) *vs. late* (L). Second, for the incubation temperature following the infection, the two levels were 15 °C *vs.* 25 °C. The full details of the procedure are explained thereafter. The factorial design allows for a classification of stressor interactions as antagonistic, synergistic or additive and is therefore useful in determining both the isolated and combined effects of stressors [14, 55, 57, 58].

### Cold treatments

Three temperature (pre)treatments were compared: two cold treatments and one control. The two cold temperature treatments were chosen based on earlier studies using similar treatments, which previously have been shown to elicit activation of both *tot* genes and other immune genes of interest [58, 72]. We exposed flies (~5 days old) to chronic cold stress (code: K; Fig. 1) by exposing groups of 10 flies (*i.e*., 10 replicates of 10 flies per treatment) to 0 °C for 6 h using 35 mL glass tubes immersed in an ice water slurry. We exposed flies (~5 days old) to acute cold stress (code: A; Fig. 1) by exposing groups of 10 flies (*n* = 10 × 10 flies) to −1.5 °C for 2 h using 35 mL glass tubes immersed in a bath of cryothermostat (VWR Collection, AP15R-30). The unstressed control flies (*n* = 10 × 10 flies) (code: C; Fig. 1) remained at the normal rearing temperature of 25 °C (±1 °C).

### Fungus treatment

**Figure 2:**
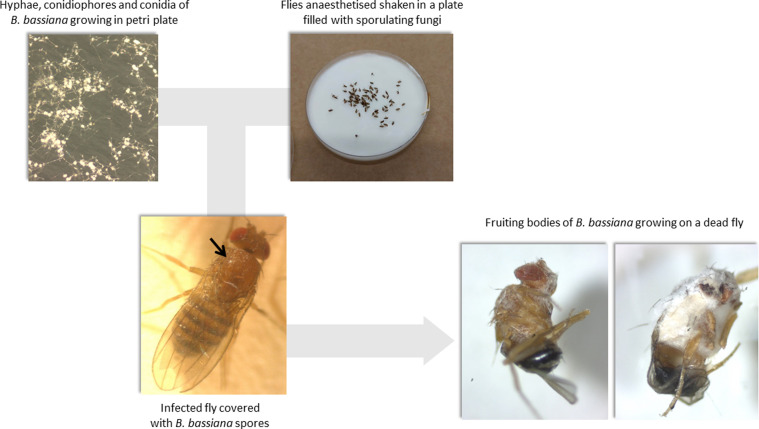
General depiction of the infection procedure showing treatment, infected individual, and subsequent mortality following infection with the fungal parasite.

The entomoparasitic fungus *B. bassiana* was chosen based on its effectiveness in earlier studies, producing significant mortality in *D. melanogaster* [8, 24, 38]. Additionally, the advantage of using *B. bassiana* is that insects may be naturally infected, avoiding the artificial wounding of tissues that is associated with manual injection (*i.e*., needle pricking), which would be necessary using other types of pathogens. The infection procedure was the same as Colinet *et al.* [8] with minor ajustments. The spores of the fungus *B. bassiana* had been stored at −80 °C in 20% glycerol before the start of the project. Briefly, 1–2 weeks before the infection, mature fungal spores (from ~ 2 week old colonies) were transposed and plated on 20 mL malt-agar plates containing 1 g Peptone (Select peptone GIBCO BRL Cat No. 30392-021), 20 g Glucose (α-D(+) glucose monohydrate ROTH Art 6780), 20 g Malt (Sigma M-0383), 15 g Select agar (Invitrogen Cat No 30392-023) and subsequently reared at 25 °C. The extraction of spores was done by adding 5 mL ddH_2_O to Petri dish plates, followed by scraping the surface using a sterile spatula, thereby creating a solution containing spores. From this solution, 200 μL of spore solution were pipetted to new Petri dish plates and smoothed out across the surface using a spatula. Before infection, the fungi were observed visually in a stereomicroscope to ensure abundant sporulation. The infection was done by transferring flies to new vials to be slightly anaesthetized with CO_2_ gas and then shaken for ca. 1 min in a Petri dish containing a sporulating fungal culture. After having checked under a stereomicroscope that all flies were well covered with spores, flies were transferred to food vials, each containing sterilized cardstock paper to increase surface area. A total of 10 replicates of 10 female flies were used for each of the three treatments groups. Pictures relating to the treatment and procedure can be found in Figure 2. Two infection groups were created in relation to the time passed since cold pre-treatments: *early* (code: E; Fig. 1) or *late* (code: L; Fig. 1) in which flies were infected 2 or 5 days post-cold treatments, for E and L, respectively. These infection times were chosen based on earlier work by Le Bourg *et al.* [36] showing that infection soon after the cold pretreatment had a stronger positive effect when using female flies. Finally, two incubation temperatures were compared by placing treated flies either at 15 or at 25 °C in incubators (MIR-154-PE, Panasonic Healthcare Co., Ltd., Wood Dale, IL, USA). The fungal virulence was expected to start after ~ 6–7 d based on a preliminary pilot study at 25 °C. After infection, mortality was scored once a day (in an interval of 24 h) and terminated when all flies died or when the dynamics of mortality had seemingly ended (typically after 14 d).

### Infection × cold experiments

These experiments aimed to investigate the possible interaction between entomoparasitic infection and the cold tolerance/response of *D. melanogaster*. Both experiments were designed to explore the effect of an infection with the fungus *B. bassiana* on the subsequent cold response. To investigate this impact, two ecologically-relevant cold tolerance proxies were utilized, namely the cold survival assay (CSA) and chill coma recovery time (CCRT). These were chosen based on effectiveness measured in earlier studies and their acceptance as ecological and scientifically relevant measures of thermal tolerance [4, 43, 61]*.* Earlier work (both prior experiments and literature data) using this fungal parasite has shown general onset of mortality following infection at ~ day 5 (at 25 °C) [8, 53, 60]. Based on this, two rounds of experiments corresponding to day 2 and 3 following infection were performed, both to ensure a minimum of 2 days recovery time following the infection procedure and to ensure that the flies were actively infected (immunity-elicited), but had not yet suffered the lethal effects of the infection. In both experiments, two treatments were compared: the infection group in which flies had been infected with the fungal parasite and a control group with non-infected flies. Both groups were kept at 25 °C in an incubation chamber (MIR-154-PE, Panasonic Healthcare Co., Ltd.) through both the rearing process and following infection to ensure stable and equal conditions. The infection procedure of the infected group was the same as that used in the previous experiments; however, as the infection procedure involved the flies being treated with CO_2_ and shaken in Petri dishes to cover them with fungal spores, a similar procedure (without any fungal parasite) was performed in Petri dish with the non-infected group to account for the possible impact of the procedure on cold tolerance.

### Cold survival assay (CSA)

A CSA is a cold tolerance measurement based on mortality dynamics of flies subjected to increasing exposure time under acute cold stress. In this experiment, we compared two groups: non-infected *vs. B. bassiana* infected*.* Flies were placed in a pre-cooled cryothermostat (VWR Collection, AP15R-30) set to −1.5 °C containing cooler fluid with ~ 50 flies contained in every glass vial. One glass vial of each treatment group was removed and transferred to new food vials, creating 5 replicates of 10 flies for each treatment and exposure duration. Flies from both groups were exposed −1.5 °C for 0, 15, 30, 45, 60, 90, and 120 min. The vials were subsequently placed at 25 °C for the rest of the experiment. Mortality scoring was done following the last treatment at intervals of 12, 24, 36, and 48 h.

### Chill coma recovery time (CCRT)

CCRT is a cold tolerance measurement using the recovery time of adult flies following chronic mild cold treatment. This recovery time materializes in the measurement of how rapidly the flies recover from a chill coma to an active mobile state following a cold treatment. This treatment was created using an ice-water slurry at 0 °C, in which the flies from both treatments were immersed within glass tubes for 8 h in a styrofoam cooler box. This treatment was chosen because it effectively induces chill coma in *D*. *melanogaster* [42]. After this, flies were collected and immediately spread on a checkered white surface with ample space between each fly, in a room at 25 °C. The time was individually noted when a fly stood on its legs and therefore was regarded as mobile/recovered (*i.e*., recovered muscle contractions). The experiment was performed twice using 50 flies of each treatment group (infected *vs.* non-infected) with only one group being used at a time to avoid cross-contamination between infected and non-infected flies. Each fly was scored individually in relation to their treatment group. After a fly was noted as recovered, it was transferred randomly to food vials with 10 flies each, creating 5 replicates per treatment group.

### *Tot* gene expression

To investigate the temporal expression of *turandot* genes following abiotic and/or biotic stress, we created five different treatments groups and one control:

1. *Acute cold* stress alone: flies exposed to −1.5 °C for 2 h.
2. *Chronic cold* stress alone: flies exposed to 0 °C for 6 h.
3. *Infection* alone: flies infected with *B. bassiana* spores for 2 days*.*
4. *Infection* × *Acute cold*: flies infected for 2 days when exposed to −1.5 °C for 2 h.
5. *Infection* × *Chronic cold*: flies infected for 2 days when exposed to 0 °C for 8 h.
6. *Control*: flies unexposed to any stress and kept at normal rearing temperature of 25 °C.

Samples were collected at two time points: 2 and 8 h following the end of treatment to give short-term insight into the induced response dynamics. The timing of when these collections were made varied with the different treatment regimens: in the single cold treatments, the collections of samples for RNA extraction were done at the two time points, immediately from the end of the cold treatments, while in the single infection treatment, the collection started 48 h after the infection procedure to ensure the flies where actively infected and to ensure a 2-day recovery period following the infection procedure. This meant that the 2-hour and 8-hour sample corresponded to 50 h and 56 h post infection. For the two combination treatments, the timing of the sampling started at the end of the cold treatments, which themselves were commenced 48 h after the infection procedure. Control flies was not subjected to any treatment; therefore, the timing of samples was of less importance. However, to avoid any confounding factors affecting the results, the control samples were collected at a similar time of day and at the same approximate age as the other treatment groups. Additionally, all flies were ensured to be of the same generation. Due to an adverse response in the treatment group of the chronic 2-hour treatment, it was not feasible to produce enough flies to create the desired amount of replicates. This treatment group was therefore omitted from the qPCR extractions.

**Figure 3:**
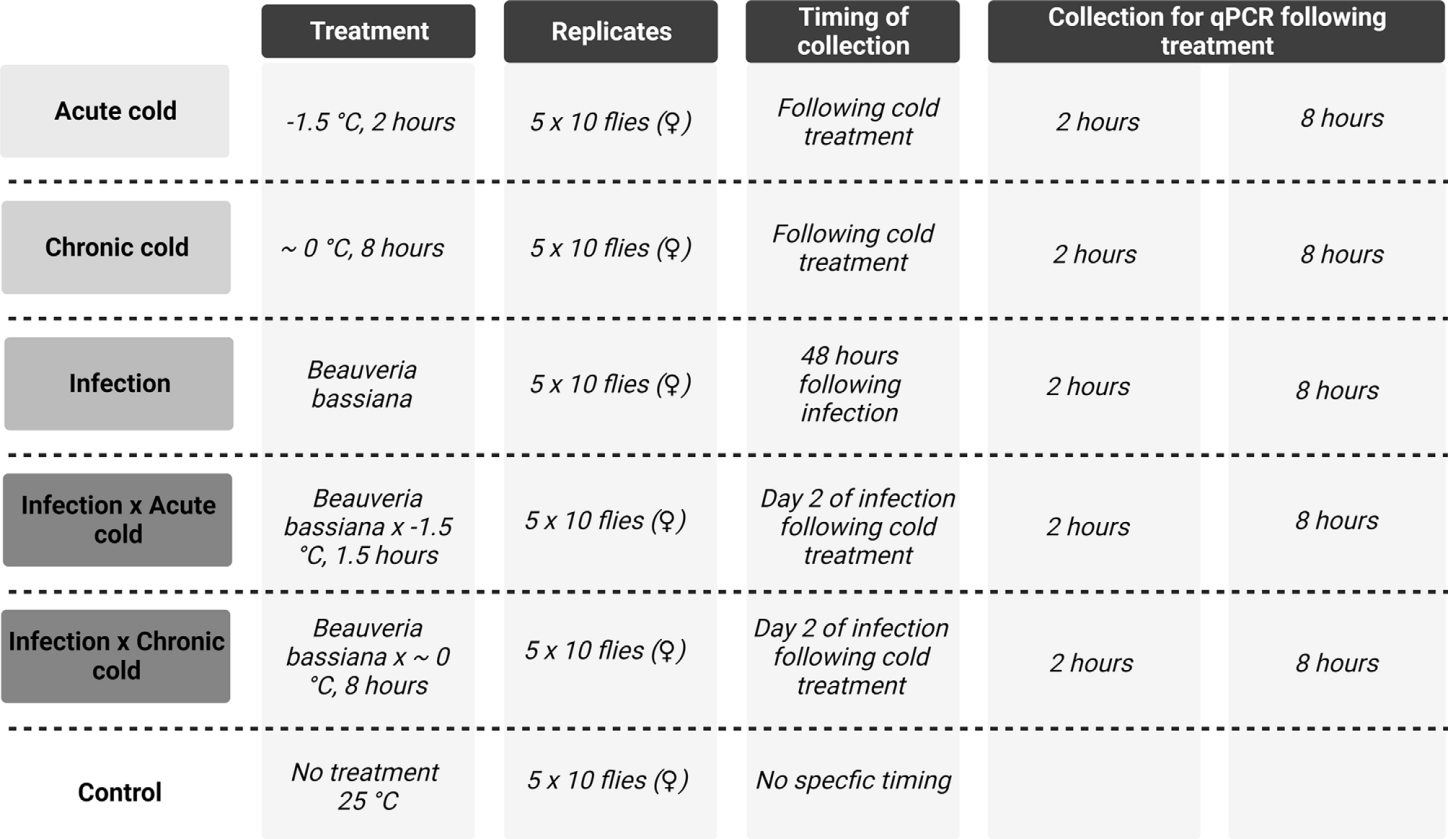
An overview of the protocol of the gene expression experiment aimed at uncovering the molecular genetic background of the cold × infection stress response. See material and methods for full descriptions of treatments.

For sampling, the flies were transferred to 1.5 mL microcentrifuge tubes and frozen immediately in liquid nitrogen and stored at −80 °C until the RNA extraction procedure. At each time point, 5 × 10 females were sampled from each treatment and time. The overall experimental design and sampling can be seen in Figure 3.

### RNA extraction and cDNA synthesis

The RNA extraction, cDNA synthesis and qPCR procedures are the same as in [19] and [39]. For each sample, replicates of 10 flies were ground to fine powder using pestles in 1.5 mL tubes placed in liquid nitrogen. Samples were mixed with 350 μL of lysis buffer (containing 1% β-mercaptoethanol) from NucleoSpin RNA extraction kit (Macherey-Nagel, Düren, Germany). RNA extraction and purification were performed using a NucleoSpin spin column, following the manufacturer’s instructions. Besides the on-column DNA digestion, any potential genomic DNA was removed using an rDNase (Macherey-Nagel). Total RNA was eluted in 40 μL of diethylpyrocarbonate-treated water. The quality and concentration of the obtained RNA was then measured by NanoDrop spectrophotometer ND-1000 (NanoDrop Technologies, Wilmington, DE, USA). An amount of 1,000 ng of total RNA was used in reverse transcription to cDNA (in 20 μL reaction mix), using Superscript III first-strand synthesis system (Invitrogen Pty Waltham, MA, USA), following the manufacturer’s instructions. The cDNA was then diluted 3-fold in diethylpyrocarbonate-treated water and stored at −20 °C until use.

### Housekeeper (HK) genes selection and qRT-PCR protocols

We targeted four *turandot* genes for qRT-PCR and tested three housekeeping (HK) genes (*RpS20*, *RpL32*, and *RpL11*) (see Table 1). The best HK gene, *RpL11*, was selected as the most stable based on the RefFinder algorithm [71]. A Roche LightCycler^®^ 480 (Roche, Basel, Switzerland) using SybrGreen I mix (Roche) was used to perform qPCRs, following the protocol described in [9]. Briefly, the PCR reaction mixture contained 10 μL in total, including 5 μL Green I Master mix, 4 μL of gene-specific primers, and 1 μL of cDNA template. The thermal cycling conditions were as follows: 1 cycle of 95 °C for 10 min, 40 cycles of 95 °C for 10 s, 60 °C for 15 s, and 72 °C for 15 s. The expression level of the target gene was transformed into fold change relative to the untreated control and normalized using the HK gene, using the ΔΔCt method [54]. To validate the specificity of amplification, a post-amplification melting curve analysis was performed as described in [9]. All qRT-PCR assays yielded specific products (*i.e*., single melting peak).

**Table 1:** Gene symbols, flybase ID and primer sequences used to assess *Turandot* (*tot*) gene expression with RT-qPCR. Ribosomal protein genes (*Rps20*, *Rpl32*, and *RpL11*) were used as housekeeping genes.

Gene symbol	Flybase ID	Forward Primer (3′ 5′)	Reverse Primer(3′ 5′)	Fragment length (bp)
*TotA*	FBgn0028396	TTCCGACGAAGATCGTGAGG	CTGGGTGCTATTGATTTTGGAGT	91
*TotC*	FBgn0044812	ATTCTGACGAGGAAAGGGAATC	CTTGGGTTCGATTGATCTTCGAT	93
*TotX*	FBgn0044810	GCAGCTTGCTAATATGCGTGT	TCGGATAGAGGAACATCTGTAGG	199
*TotM*	FBgn0031701	TCACAGAAAAACAGCGCCTAT	ATCGTAGAAAGTGACCAGGCT	98
*RpS20*	FBgn0019936	TGGTGATGCGAAGGGTCTTG	CCGCATCACCCTGACATCC	134
*RpL32*	FBgn0002626	TATTCCGACCACGTTACAAGAAC	GCTTCAAGGGACAGTATCTGATG	306
*RpL11*	FBgn0013325	GTATTCGCCGTAACGAGAAGAT	GATGCCGAAACCGAAGTTGC	146

### Statistical analysis

All statistical analyses were done in R-studio (2023.03.1, Build 446) using R version 4.2.2 [56]. Almost all data were from factorial repeated measure designs, which lend themselves well to the use of generalized mixed effect models. Before the creation of any models, thorough data exploration was performed, as well as subsequent systematic check of assumptions of the models to ensure the robustness of the statistical analysis. The fit of models was generally investigated using Akaike information criterion (AIC) based methods [2].

To investigate the effects of the three predictors: Treatment (*Acute*, *Chronic*, and *Control*), Infection time (*Early vs. Late*) and Temperature (25 *vs.* 15 °C) and on the binary response variable (Dead/Alive), we built a generalized mixed effect model (GLMM) using the “glmer” function from the “lme4” package [5]. We utilized the GLMM here, to account for the non-normal binomial distribution of our data, which is produced by the data being proportional data expressed only between 0 and 1. In the function, this is accounted for by using the “binomial loglink” family option. Additionally, the mixed effect part is included to incorporate the potential variance introduced in the model by the repeated measurements (*i.e*., repeated measures on the same individuals across time points) on vials containing the same flies. Therefore, to avoid any pseudo replication effects on the model, the vial IDs were included as a random factor. Subsequently, after AIC-based model performance testing starting on a model including all possible second-degree interactions, the final model became:

$$\mathrm{glmer}\left(\left(\frac{\mathrm{Dead}}{\mathrm{Alive}}\right)∼\;\mathrm{Treatment}+\mathrm{Temperature}+\mathrm{Infection}\;\mathrm{time}+\;\mathrm{Day}\;+\mathrm{Treatment}:\mathrm{Temperature}\;+\;\mathrm{Treatment}:\mathrm{Infection}\;\mathrm{time}\;+\mathrm{Treatment}:\mathrm{Day}\;+\;\mathrm{Temperature}:\mathrm{Infection}\;\mathrm{time}+(1|\mathrm{ID})\right).$$

**Figure 4:**
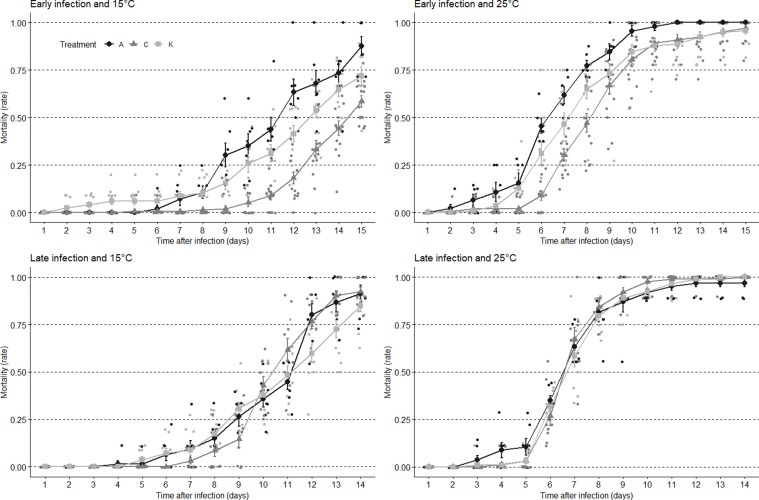
Temporal mortality dynamics of flies infected with *B. bassiana* subsequent to pre-treatments: *acute* (A) −1.5 °C/2 h, *chronic* (K) 0 °C/6 h, or *control* (C) 25 °C. Data are presented according to the timing of infection post-cold treatment (*early* 2 days *vs. late* 5 days, top *vs.* bottom) and to the incubation temperature (15 *vs.* 25 °C, left *vs.* right). Data are mean + *SE* (*n* = 10 × 10 flies).

Additionally, four individual GLMMs were created to investigate the direct effect of treatment interacting with time (*i.e*., sampling day) within each temperature and infection time combination (*i.e*., two models for *early* 15 and 25 °C and two models for *late* 15 and 25 °C). The aim of these sub-models was to test whether temporal dynamics of infection differed according to treatment, which would materialize as a significant interaction term (Fig. 4). These models were built as follows: $\mathrm{glmer}(\left(\frac{\mathrm{Dead}}{\mathrm{Alive}}\right)\;∼\;\mathrm{Treatment*Day}+(1|\mathrm{ID}))$. Furthermore, to examine the difference between the treatments within these groups, pairwise comparisons using *t*-tests with pooled standard deviation (*SD*) were used. Here, the *p*-values were adjusted using the Bonferroni correction method to account for multiple comparisons.

For CCRT, data were “time to recovery” (min) and event (coma *vs.* awoken) according to two treatments (infected *vs.* non-infected) on two separate days of the infection stage (day 2, day 3). This event type data, signified by producing 0 or 1 if an event has occurred or not, lends itself well to be analyzed using “Surv” and “survdiff” functions of the “survival” package, which are designed to work with this type of data and can be utilized to produce log-rank and Wilcoxon tests to assess differences of resulting CCRT curves among the treatments. To supplement these tests, a GLM model was produced, which opened up the ability to further investigate the individual effects of the predictors using a deviance analysis (ANOVA). As time-to-event data normally result in gamma distribution, the Gamma(link=“log”) function was used to specify this distribution. Treatment and Day were both treated as factorial variables. The final model produced was:

$$\mathrm{glm}(\mathrm{Time}\;∼\;\mathrm{Treatment}\;*\;\mathrm{Day},\;\mathrm{family}\;=\;\mathrm{Gamma}\left(\mathrm{link}=“\log”\right).$$

For the CSA, a similar statistical approach as with the cold × infection experiment was used. Our aim was to investigate the effect of four predictors: Treatment (infection *vs.* non-infection), Cold treatment time (15, 30, 45, 60, 90, 120 min), Infection stage (day 2 *vs.* day 3), and Time of observation (12, 24, 36, 48 h) on the response variable Dead/Alive.

Again, we accounted for both the binomial distribution of the mortality data as well as incorporating a random factor introduced by the repeated measurements by using a GLMM model with the “glmer” function from the “lme4” package. Model selection was again done in relation to AIC values. Here, the best model included two second-degree interactions; however, after investigating the VIF values, an abnormal amount of multicollinearity was found in these interactions, resulting in them being removed from the final model. The model therefore resulted in:

$$\mathrm{glmer}\left(\left(\frac{\mathrm{Dead}}{\mathrm{Alive}}\right)\;∼\;\mathrm{Treatment}+\mathrm{Time}+\mathrm{Infection}\;\mathrm{stage}+\mathrm{Time}\;\mathrm{of}\;\mathrm{observation}+(1|\mathrm{ID})\right).$$

For gene expressions, the resulting fold change values of the different treatment groups relating to the different genes were statistically investigated using an ANOVA model performed on a “lm” formula: lm(fold change ~ treatment). To account for non-normality of the data, we log2 transformed the *fold change* variable, which markedly improved the model in relation to the assumptions of a linear model. To explore the differences among the multiple treatments, Tukey-tests from the “emmeans” package were performed. Additionally, *t*-tests were performed investigating if the fold change values were significantly different from the mean of the control.

## Results

### Cold × infection experiments

For the cold × infection experiments, we were interested in elucidating the potential cross-protective effects induced by two cold treatments on the actual survival of *D. melanogaster* infected with the entomoparasite fungus *B. bassiana.* The analyses showed a significant effect of all the predictors on the mortality percentage following the infection (as seen in Table 2), meaning that we do see a significant impact of treatments, temperature, and infection time regimes. To further investigate how these conditions affected the relationship between the treatments and mortality percentage, four different GLMMs were created using the same ANOVA approach (Table 3). Here, across all combinations, we detected a significant effect of the variable “Day”, translating the increase in mortality with time after infection (see Fig. 4). Yet, these temporal increases in mortality varied according to treatments, as denoted by significant interactions (see Table 3 and Fig. 4). Additionally, we found a significant main effect of treatment in both the early infection/temperature combination but not in the late infection/temperature combination, meaning that we no longer had an established effect of treatment on the mortality percentage at the late infection stage (see Table 3 and Fig. 4). Contrary to expectations, cold pre-treatment did not provide protection to fungal parasite but rather made the flies more susceptible to infection, and this was only apparent when cold treatment preceded infection by a few days (*i.e*., *early* conditions) (Fig. 4). The differences among treatments were further investigated using multiple comparison tests with Bonferroni correction. In the *early* infection group at 15 °C, a highly significant difference was observed between *acute* and *control* treatment groups (*p* = 1.6 × 10^−7^), indicating that these groups had distinct effects on mortality. However, no significant difference was found between *acute* and *chronic* treatment groups (*p* = 0.34). Similarly, a highly significant difference was observed between *control* and *chronic* treatment groups (*p* = 3.1 × 10^−4^). In the *early* infection group at 25 °C, a significant difference was detected between *acute* and *control* treatment groups (*p* = 0.04), indicating distinct effects of these pre-treatments. However, no significant differences were found between *acute* and *chronic* (treatment groups *p* = 0.40) or between *control* and *chronic* treatment groups (*p* = 0.73). In the *late* infection group at 15 °C and 25 °C, no significant differences were observed between any of the treatment groups (*p* > 0.05).

**Table 2:** ANOVA table for the cold × infection treatment groups. Analysis produced using the “car” package, Type II Wald chi-square tests.

Variables (full model)	*χ* ^2^	*Df*	Pr (>*χ* ^2^)
Treatment	39.54	2	2.61 × 10^−9^
Temperature	1944.20	1	< 2.2 × 10^−16^
Infection time	270.27	1	< 2.2 × 10^−16^
Day	3639.20	1	< 2.2 × 10^−16^
Treatment: Temperature	85.70	2	< 2.2 × 10^−16^
Treatment: Infection time	176.11	2	< 2.2 × 10^−16^
Treatment: Day	83.93	2	< 2.2 × 10^−16^
Temperature: Infection time	57.01	1	4.26 × 10^−14^

**Table 3:** ANOVA tables for each infection/temperature group. Analysis produced using the “car” package, Type II Wald chi-square tests.

	Variables	*χ* ^2^	*Df*	Pr (>*χ* ^2^)
*Early* infection, 15 °C	Treatment	45.38	2	1.4 × 10^−10^
	Day	701.84	1	2.2 × 10^−16^
	Treatment: Day	39.82	2	2.3 × 10^−9^
*Early* infection, 25 °C	Treatment	22.04	2	1.6 × 10^−5^
	Day	1154.40	1	2.2 × 10^−16^
	Treatment: Day	14.12	2	8.6 × 10^−4^
*Late* infection, 15 °C	Treatment	0.59	2	0.74
	Day	772.46	1	2.2 × 10^−16^
	Treatment: Day	34.36	2	3.5 × 10^−8^
*Late* infection, 25 °C	Treatment	0.94	2	0.62
	Day	666.71	1	2.0 × 10^−16^
	Treatment: Day	30.31	2	2.6 × 10^−7^

### Infection × cold experiments

**Figure 5:**
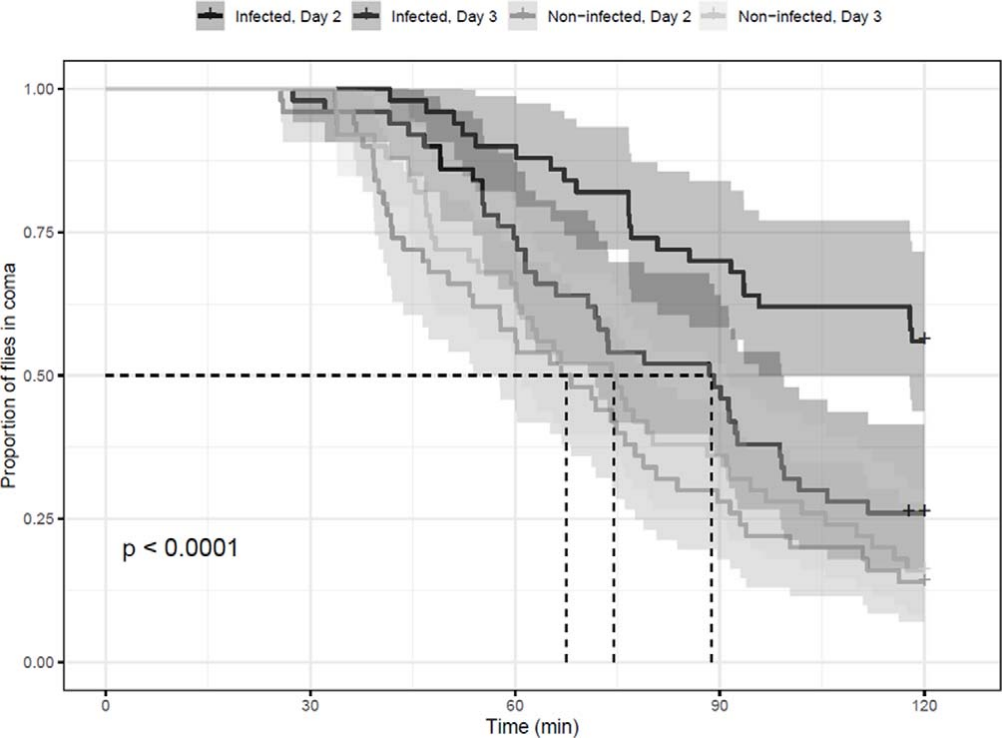
Proportion of flies in coma in relation to time for the different treatments indicated on top, with confidence intervals and *p*-values indicating the statistical significance of differences between the recovery curves. “Day” signifies the time following infection, while non/infected signifies the treatment (*n* = 50 flies).

**Figure 6:**
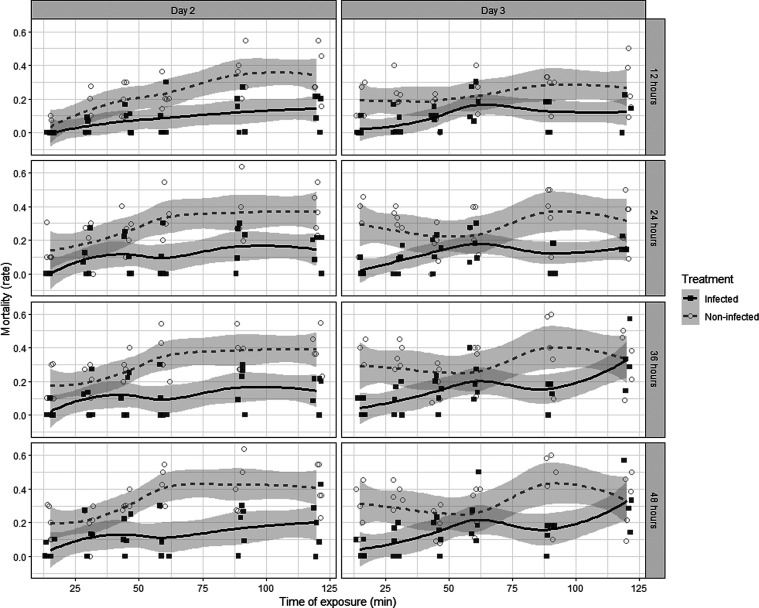
Mortality rate following acute cold shock according to the three predictors: cold exposure duration on *x*-axis (Time), Day of infection (panel Day 3 *vs.* Day 2) and Treatment (infected *vs.* control: clear *vs.* dark gray color). Confidence intervals of the fitted loess function (*y* ~ *x*) are shown (*n* = 5 × 10 flies).

For the infection × cold experiments, our aim was to test the potential cross-protective effect of parasitic infection by *B. bassiana* on the cold tolerance of *D. melanogaster* flies. This was done using two different proxies for cold tolerance, namely CRRT and CSA. Temporal dynamics of CCRT varied according to treatments and infection state (log-rank test, *p* = 2.0 × 10^−6^) as illustrated in Figure 5, showing the experimental time on the *x*-axis and the proportion of flies in coma on the *y*-axis. Because *post hoc* analyses are not available and are not reliable with log-rank tests, additional tests such as General Linear Model (GLM) are required to determine where the differences among groups lie [34]. The GLM with a gamma distribution for modeling time-to-event data showed that variable “Treatment” exhibited a significant effect on the time to recovery (χ^2^ = 19.66, *df* = 1, *p* = 9.2 × 10^−6^), suggesting that different pre-treatments had a notable impact on the timing of recovery from chill coma. Similarly, the variable “Day” demonstrated a significant influence on the recovery outcome (χ^2^ = 6.72, *df* = 1, *p* = 0.009). However, the interaction between “Treatment” and “Day” did not show a significant effect on the recovery outcome (χ^2^ = 1.39, *df* = 1, *p* = 0.24). This implies that the relationship between treatment and the timing of the event did not depend on the specific day of infection. For the CSA, we observed a very clear positive effect of infection on subsequent cold tolerance, with consistent reductions in cold-induced mortality in infected flies compared with control flies across almost all sampling periods, exposure durations and days of infection (Fig. 6). The analysis of deviance revealed significant effects for the variables Treatment, Time and Time of observation on mortality proportion as seen in Table 4.

**Table 4:** ANOVA table for the Cold × infection treatment groups. Analysis produced using the “car” package, Type II Wald chi-square tests.

Variables	*χ* ^2^	*Df*	Pr (>*χ* ^2^)
Treatment	84.27	1	2.2 × 10^−16^
Time	91.22	1	2.2 × 10^−16^
Infection stage	2.32	1	0.13
Time of observation	27.70	1	1.4 × 10^−7^

### *Tot* genes expression

**Figure 7:**
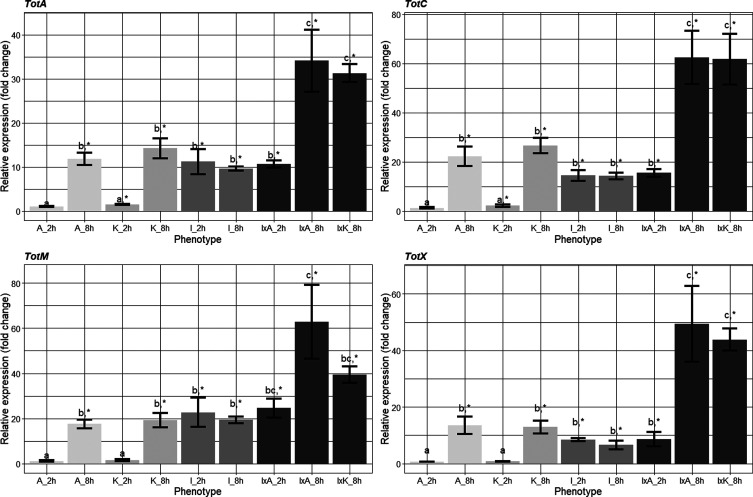
Mean fold changes (*n* = 5) relative to control in the four different *tot* genes (*TotA*, *TotC*, *TotM*, *TotX*). Significant differences, resulting from Tukey post-hoc tests, are indicated with different letters. The symbol (*) indicates when mean values were different from the control. The treatment groups are from left to right: Acute 2 h, Acute 8 h, Chronic 2 h, Chronic 8 h, Infection 2 h, Infection 8 h, Infection × acute 2 h, Infection × acute 8 h, Infection × chronic 8 h.

Differences in normalized relative expressions (*i.e*., fold change) of the four different *tot* genes according to treatments are shown in Figure 7. As indicated in Table 5, there were differences among treatments for all tested genes. In general, *tot* genes were upregulated 8 h after cold stress (*A* and *K*) and also after infection alone, with fold changes around 10 to 20-fold. In all genes, expression levels increased distinctively, with fold changes reaching 35 to 60-fold, when cold stress (*A* and *K*) was applied in flies that had been previously infected with the parasite (Fig. 7). Tukey-tests were performed to investigate whether the fold changes induced by the different treatments were significantly different from one another. Here, three distinct groups emerged across all the *tot* genes: First, the two 2-hour cold treatments (*A* and *K*) exhibited similar effects, both yielding values close to 1. Second, the treatments Infection (2 and 8 h), infection × acute cold (2 h) and the two 8-hour singular cold treatments, formed another group. These treatments resulted in values ranging from approximately 10 to 20-fold increase. Third, the two 8-hour combination treatments had the most significant impact on gene expression, which therefore denotes their own grouping with increases up to 60-fold. These three groups generally reflect the upregulation patterns depicted in Figure 7, showing a general increase of gene expression with time, as all the 8-hour treatments generally had a higher fold-change in relation to their 2-hour counterparts. An exception was the infection regimes, which both had similar fold changes at the sampled timepoints. Additionally, *t*-tests were performed to investigate whether the fold changes induced by the different treatments were statistically significant from the control group (see Fig. 7). For *totA*, a significant difference was found in all treatments, except the 2-hour acute cold treatment (*t* = 0.25, *df* = 2, *p* = 0.82). For *TotX*, a similar outcome obtained with the 2-hour acute treatment (*t* = −4.17, *df* = 2, *p* = 0.052) was found to yield non-significant values, while the rest of the treatments were significantly different from the control. For the *TotM* and *TotC* genes, we found similar significant values for all treatments apart from the 2-hour acute treatment (*TotM*: *t* = −0.07, *df* = 2, *p* = 0.94; *TotC*: *t* = 1.37, *df* = 2, *p* = 0.30) and 2-hour chronic treatment (*TotM*: *t* = 1.45, *df* = 2, *p* = 0.28; *TotC*: *t* = 3.16, *df* = 2, *p* = 0.87) that were non-significant.

**Table 5:** ANOVA tables for each *tot* gene testing effect of treatment on log2 fold change expression.

*Gene*	*Df*	Sum sq	*F*-value	Pr (>*χ* ^2^)
*TotA*	8	124.16	69.52	2.2 × 10^−16^
*TotC*	8	163.54	86.86	2.2 × 10^−16^
*TotM*	8	173.33	36.95	4.0 × 10^−14^
*TotX*	8	168.65	48.79	8.0 × 10^−16^

## Discussion

### Cold × immune

We found no support for the first hypothesis, which assumed an interaction between exposure to cold stress and a potential subsequent cold-induced infection tolerance. We did not observe any cross-protective response induced by either of the two cold pre-treatment regimens consisting of acute or chronic cold. These results overall mirror the results of earlier studies using similar cold treatments [59, 75]. Here, neither of the cold exposures elicited an increase in actual immunity, though a significant increase in potential immunity was observed by activation/upregulation of central immune genes.

An interesting dynamic elucidated by this study was the significant difference found between the two cold pre-treatments and the control group concerning their impact on mortality rates in the early- and late-infection groups. Cold pre-treated flies from the early infection group had significantly higher mortality in comparison to the control flies, while no significant difference between the three treatments was found in the late infection group (as observed in Fig. 4). This could indicate that cold pre-treated flies are more susceptible to the entomoparasite infection in the early stages following the cold treatment. This would align with earlier work suggesting a negative relationship between cold and immune capabilities [7, 20, 33, 52]. However, this largely pertains to a long-term impact of cold exposure and does not widely take into account within-generation adaptative plasticity. Since we found a change between the early and late infection stage, this effect is more likely to be related to the damage caused and subsequent recovery from the cold stress treatments. All flies, from both cold treatments, were put into chill coma. Transcriptomic profiles have shown that this comatose state is detrimental to survival and induces a cold stress response [41, 59, 75]. Hence, we can safely assume that comprehensive damage was induced in the flies. Both recovery from chilling injury [46] and immune responses [22] are metabolically costly in the insects, which could explain the initial decrease in immune capabilities following the cold stress as representing a trade-off between recovery from cold exposure and immune activity, with early infected flies still being in an active recovery phase following the cold stress, while the chill recovery had been completed in the late infection group.

In addition to the effect of treatment and infection time, another noteworthy effect was the significant positive effect of incubation temperature on temporal mortality responses. For all infection times and treatments, the general pattern showed faster mortality in infected flies incubated at 25 °C (Fig. 4). This suggests that the host-pathogen effect was greatly determined by the environmental temperature at which this interaction took place. Earlier work investigating *B. bassiana* found that virulence (both in terms of overall mortality and dynamics) was indeed affected by environmental temperature [28, 29]. This suggests that the 25 °C incubation following infection was closer to the thermal optimum of the fungi, which has previously proved decisive for its distribution patterns and infection efficiency [21, 32]. This indicates that the host-pathogen relationship is not only impacted by the host’s thermal preference and performance, but also by the pathogen’s thermal preference and performance. Hence, the outcome of this interaction lies in the trade-off between how temperature affects the ability of the pathogen to colonize a host, versus the effect of temperature on the ability of the host to defend itself [70]. This is one of many considerations that needs to be taken into account when performing biotic stress experiments in interaction with abiotic factors, such as temperature.

As explained, the physiological responses to cold differ both among the types of cold exposure and species of insects [74], with prolonged chilling (*i.e*., chronic stress) leading to widely different types of damage in the organism than acute intense cold [15, 46, 73]. This means that even though we did not produce any cold-induced immunity to the entomoparasite, this does not necessarily mean that the mechanism for this does not exist in the organism. As previously proposed, the immune response following cold exposure could be based on a necessity for the response to repair damage following cold exposure [62]. It can therefore be argued that this realized immune response is specific for only some types of damage following cold stress and that the treatments used in this study were simply not adequate in inducing this response. For example, this could be the reason behind the induced actual immunity produced by Le Bourg *et al.* [36] using a different cold treatment regime. In their study, a repeated cold stress regime using mild stress (~ 0 °C) successfully induced a cross-protective response towards fungal infection. Moreover, the intensity of damage (*i.e*., intensity of cold stress) may also be of significance. For instance, multiple studies have proposed that the immunity-related genes like *turandot* genes respond specifically to severe cold stress [3, 59]. This means that both the type and intensity of cold stress are confounding factors that must be considered, when evaluating the results of cold × immune experiments.

### Immune × cold

Regarding the immune × cold interaction, we hypothesized that we would observe an actual cross-protective effect of pathogenic stress inducing an increased cold tolerance in two types of cold tolerance measurements: chill coma recovery time and acute cold tolerance. In relation to the study of the impact of infection on the chill coma recovery, we did not find that infected flies produced a quicker recovery time compared to non-infected flies. Contrarily, we did see non-infected flies having a significantly quicker recovery either at day 2 or day 3 of the infection stage as shown in Figure 5. These results generally align with Linderman *et al.* [40], who tested the impact of an infection with two strains of pathogenic bacteria, *L. monocytogenes* and *S. pneumoniae*, on the chill recovery of *D. melanogaster.* Here, the infected strain was significantly slower and less likely to retain a standing position following a cold regime. As with the previous experiment, this could be because of a conflict between the energy allocation already being used for the immune response and thereby overwhelming the protective mechanisms [23]. This notion is supported by the variable “Day” having a positive significant effect on the recovery time, indicating that as we get further in the infection process, the recovery time increases. This could be seen as an accumulation of damage and a depletion of energy reserves associated with the dynamics of the infection, as both parameters have earlier been linked to the chill coma recovery time [25, 40]. In general, these results provide evidence in line with earlier findings, showing no general relationship between cold stress/response and immune stress/response theorized as being part a general stress responses in the organism. These outcomes both differ from what was expected in relation to the hypotheses and some literature emphasizing the existence of such an interaction [20, 36, 59, 62].

While an adaptive cross-tolerance response was not found in the previous experiment, when it comes to the CSA and the induced cold resistance following infection, we found that infected flies survived at a significantly higher level across all sampling times, cold treatment durations, and infection stages, as observed in Figure 6. This indicates a potent cold resistance response in the flies as a result of the infection status and therefore strongly suggests a cross-protective effect, as we had hypothesized. These results evidently stand in stark contrast to the negative effect of the infection found in relation to the chill coma recovery time and possibly insinuates the fundamental differences between the chronic and acute cold treatments. As explained earlier, these two types of cold stress are known to induce different types of damage, which potentially could mean that the type and intensity of damage could elicit vastly different responses in the organism [15, 46, 73, 74]. Earlier research done by Salehipour-Shirazi *et al.* [59] showed increased immunity following acute cold stress, but not chronic cold stress, indicating a fundamental difference in the underlying genetic architecture of the responses. This idea is furthermore supported by the divergent transcriptomic responses found between chronic, acute, and repeated cold stress regimes [75], indicating that despite a general activation of the immune response following cold, distinct groups of genes with non-overlapping activation, could explain distinct functional significance. Since, in this case, we found distinct patterns of cold tolerance following infection (with CCRT showing lower cold tolerance and CSA higher cold tolerance), we could deduce that the induced immune genes of the infection have more specific overlap with the type of damage induced by the applied acute cold stress. This is in line with Rodgers and Gomez Isaza [57], who redefined the cross-protective response to chronic stressors as *cross-acclimation*, based on the general physiological remolding, following the chronic exposure. However, this notion has not yet been used in relation to experimental work, and the constitution of a chronic stressor is inherently different among organisms, making it somewhat inapplicable in relation to our study.

As a whole, our results suggest that it would be unlikely for the cold-immune interaction to be a by-product of a larger general stress response, but rather a more specific response driven by the overlap of damage induced by cold shock injury and pathogenic infection, *e.g.*, apoptosis or membrane phase transitions [15, 37, 73]. Thus, the response would be driven via direct signals from apoptotic or necrotic cells [50]. These suggested mechanistic connections have relevance in unraveling the evolutionary history of cold-immune interactions. As the cross-protective response is linked to shared signaling in relation to tissue/cell damage, it could be proposed that the mechanism behind said response is driven by cross-talk among the pathways. This would align with immune interactions being activated by multiple other stress signals in *Drosophila* also implying the existence of cross-talk mechanisms, as previously postulated [12, 62]. Sinclair *et al.* [62] proposed that the selective pressure driving this interaction could be linked to adaptive pre-emptive activation of immunity linked to damage of the tissue or gut (*e.g.*, induce by cold stress), leading to an invasion of the hemocoel by microbiota.

### *Tot* genes expression profiles

As pertaining to the hypothesized relationship of a actual immune/cold resistant response and a subsequent concurrent upregulation of *tot* genes, we found a general upregulation of *tot* genes for all treatments, except the early single cold treatments (*i.e*., 2 h post cold), which mostly showed non-significant upregulation relative to the control treatment. We therefore have support for the hypothesized notion that *tot* genes are both cold- and pathogen-responsive genes, as also found in earlier studies [16, 18, 58, 72]. As seen in Figure 7, we found the strongest upregulations for all *tot* genes in the combined treatment groups at the 8 hour time point (generally 2- to 3-times higher than the singular treatments). This implies a formidable direct response of these genes to the synergetic effect of cold stress and pathogenic infection. This finding gains additional significance considering the higher survival rate observed in flies subjected to the infection × acute cold treatments. The synergetic nature of the expression of *tot* genes, as uncovered in this study, is consistent with earlier findings implying that *tot* genes generally require more severe stress for activation in relation to other stress related genes like *HSP* [16, 17]. Given that severity appears to have a substantial impact on gene activation, this might suggest a mechanistic purpose for the *tot*-response, particularly geared towards scenarios where multiple stressors concurrently affect the organism, as is commonly expected in the natural environment. This would align with the hypothesized cross-protective nature of the genes and potentially suggest their ecological relevance as part of a general stress response in the organism [3, 51].

The temporal dynamics of *tot* genes expression is generally understood as exhibiting a slower, but more persistent expression relative to HSP response, which normally are regarded as a molecular stress response peaking early after induction [3, 11, 16, 17]. Our results corroborate this notion, as we observed a discrepancy between the 2-hour and 8-hour time points in both the single cold treatments and the acute × infection treatment, with peak expression found at the later time points. These dynamics support the proposal of *tot* genes as part of a more long-term stress response. The same pattern is not found in the infected treatments. Here, we largely see a similar effect of the infection in at both the early and the late time points. However, this is most likely due to the 48-hour incubation the flies had following the infection procedure, which with all likelihood made the difference in infection load between the two time points benign. The incubation period was mainly done for logistical purposes and to ensure an equal age between all treatment groups but should be avoided in potential future studies using the same experimental design.

In conclusion, this study gives insight into the complex interaction between the effect of cold and pathogenic stress and the subsequent plastic responses. We found no cross-protective immune response on mortality propensity in cold treated *D. melanogaster* flies, when infected with the entomoparasite *B. bassiana*. Conversely, we did find higher susceptibility to infection in flies, when they were infected early following the cold treatments. Similarly, no cross-protective effect was observed between infection and the cold tolerance measurement of chill coma recovery time. Here, infected flies had a generally slower recovery time in relation to non-infected flies. In contrast, we did find a clear-cut cross-protective effect in relation to the acute cold survival, where infected flies in general showed a higher survival propensity in relation to the non-infected flies, indicating an actual higher cold resistance in the infected group. For the results investigating the activation and following upregulation of *tot* genes, we found a general response to both cold and infection treatments, with the combination treatments producing the strongest responses. This suggests that the synergetic property of the combined stress has an enhancing effect on the activation of *tot* genes. These results should be regarded as a steppingstone for the advancement of our understanding of the functional impact of the cross-protective effects produced by genes like the *tot* family. In general, more research is needed, especially in the light of expected climate change, potentially affecting the eco-immunology of small insects.
